# A global perspective on assisted reproductive technology fertility treatment: an 8-country fertility specialist survey

**DOI:** 10.1186/s12958-015-0131-z

**Published:** 2015-12-09

**Authors:** Céline Audibert, Daniel Glass

**Affiliations:** Deerfield Institute, Route de la Corniche 3a, 1066 Epalinges, Switzerland; Deerfield Institute, New York, USA

**Keywords:** Assisted reproductive technology (ART), In vitro fertilization (IVF), Infertility, Live birth rate, Pregnancy rate

## Abstract

**Background:**

Procedures that may optimize success in achieving live births from assisted reproductive technology (ART) continue to be examined. Not yet considered are the perspectives of fertility specialists regarding important developments in the fertility treatment field, current unmet needs, and anticipated future advances. In the current study, an 8-country survey of fertility specialists was conducted to provide a comprehensive, global depiction of fertility treatments across different regions.

**Methods:**

Fertility specialists from France, Germany, Italy, Spain, the United Kingdom (UK), the United States (US), China, and Japan were invited to participate in an online survey. Participants were eligible if they personally managed ≥25 patients/month who were experiencing difficulty conceiving, and if they had performed ART fertility treatment with ≥1 patient in the previous month. Quantitative questions addressed the number of patients seen, main infertility causes, number of cycles performed, ART procedure type, and ART outcomes. Qualitative questions covered diagnostic trends, unmet needs, important advances, and expected future developments.

**Results:**

The number of fertility specialists who completed the survey included 29 in France, 33 in Germany, 23 in Italy, 38 in Spain, 34 in the UK, 91 in the US, 50 in China, and 65 in Japan. Patient volume increased over the prior 2 years according to 67 % (242/363) of the fertility specialists. As expected, ART outcomes all declined with age in all countries. ART outcomes varied by country, with the highest implantation, pregnancy, and live birth rates reported by fertility specialists in the US and China and the lowest rates reported in France and Italy. The most frequently reported unmet needs in fertility treatment were financial coverage, improved implantation rate, and egg donation. Most frequently named future advancements expected to change the fertility treatment field included improved embryo selection through imaging and/or metabolomics, improved embryo implantation rate, and use of preimplantation genetic diagnosis.

**Conclusions:**

This study, which follows a rigorous survey methodology, elucidates the current state of fertility specialists’ practices and perspectives on the global fertility treatment field, which highlights differences and similarities among countries. This research may inform further studies and procedural developments that might better improve and standardize ART.

## Background

Considerable progress continues to be made in assisted reproductive technology (ART) fertility treatment since the birth of the first “test tube baby”, Louise Brown, in 1978 in the United Kingdom (UK), followed by other live births in Australia (1980), the United States (US) (1981), and in Sweden and France (1982) [1, 2]. However, fertility treatment remains a complex field that involves many different dimensions over the course of patient treatment, such as hormonal treatment to produce eggs, retrieval of eggs and sperm, varied fertilization techniques, such as in vitro fertilization (IVF), intracytoplasmic sperm injection (ICSI) and intracytoplasmic morphologically selected sperm injection (IMSI), use of one’s own or donor eggs, fresh versus frozen embryos, number of embryos to transfer, and preimplantation genetic diagnosis (PGD) [2]. Additionally, variations in fertility treatment exist among countries, such as illegality of donor IVF cycles in Germany; illegality of PGD and preimplantation genetic screening (PGS) in France; and illegality of embryo storage in Italy until 2009, mandating a requirement that all generated embryos be implanted.

Fertility specialists are continually examining ways to optimize the techniques and processes within the fertility treatment field to improve success in achieving live births. Recent targets of interest have included analyzing procedures for ovulation induction and triggering final oocyte maturation [3–6], improving the prediction of ovarian response to stimulation [7, 8], determining optimal duration of co-incubation of gametes [9], assessing endometrial receptivity and implications for the use of fresh versus frozen embryos [10, 11], examining embryo culture techniques [12], monitoring embryo development through new techniques such as time-lapse embryo monitoring [13], and improving embryo selection for transfer [14]. Despite numerous improvements in fertility treatment, the number of factors that have an impact on fertility make fertility difficult to control and the desired outcome of a live birth cannot be guaranteed. Fertility specialists are continuously looking for ways to improve pregnancy rate (PR) and live birth rate (LBR) in patients.

Large ART fertility treatment registries exist through the European IVF Monitoring Consortium on behalf of the European Society of Human Reproduction and Embryology (ESHRE) in Europe [15] and the Centers for Disease Control and Prevention (CDC) in the US [16]. Although the registries provide comprehensive data on the current state of ART in Europe and the US, they have some important limitations. The registry reports are published with some delays, with the currently available publications reporting 2010 data in Europe, and 2012 data in the US. Additionally, the registry reports are based exclusively on quantitative data. There are no known publically available qualitative data describing the perceptions of fertility specialists regarding current unmet needs or the important developments in the field of fertility treatment. For the ESHRE report, the method of reporting data to the registry is not standardized and consequently there is variability among countries in the information reported.

ART, and more specifically IVF, continues to change and further develop with new technologies. However, many questions remain, including: What have been the true innovations in the field of fertility treatment, and how have they affected the treatment of patients? What are the differences per country in fertility treatment? Where are fertility treatments headed in the future? In the current study, an 8-country survey of fertility specialists was conducted with the aim to provide a comprehensive depiction of fertility treatments across different regions. Using a standardized survey-based approach, the goal of the research was to provide both quantitative data and qualitative perceptions from fertility specialists regarding which developments have been most important in the field of fertility treatment within the past 30 years, the current unmet needs in fertility treatment, and which anticipated improvements will be “game changers” in fertility specialty practices in the coming years.

## Methods

### Study participants and design

Fertility specialists from the European Union Five (EU5, which includes France, Germany, Italy, Spain, and the UK), the US, China, and Japan were invited to participate in an online survey aimed at understanding current management of women experiencing difficulty conceiving. The criteria for the selection of these countries included having a high number of patients who undergo ART, the representation of a broad spectrum of fertility treatment regulations and practices across the included countries, and high access to the internet within each country. In the EU5 and US, a sample frame of 3975 fertility specialists was established by assembling physicians from public sources, such as the ESHRE annual ART reports and the CDC web site, that list fertility clinics [17]. For China and Japan, no lists of fertility clinics were found. For that reason, an external panel provider, M3, was used. M3 had access to 39,930 endocrinologists, obstetricians/gynecologists, embryologists, and reproductive health specialists in China and 11,366 in Japan. Practice specialty was self-referenced by survey respondents, but did not include the fertility specialization, which was why the sample frame of physicians in China and Japan was larger than in the other participating countries. In China and Japan, the verification of a participant’s fertility specialist credentials was achieved through the eligibility screening criteria of the study. The physician sample was randomized and no other factors applied to influence completion patterns. In the EU5 and US, all 3975 fertility specialists contained within the sample frame were invited to participate in the survey by e-mail or postal mail. In China and Japan, endocrinologists, obstetricians/gynecologists, embryologists, and reproductive health specialists were invited by e-mail only. Fertility specialists were eligible to participate if they personally managed ≥25 patients per month who were experiencing difficulty conceiving, and if they had performed ART with ≥1 patient in the previous month. Participants were offered an industry-standard honorarium as compensation for their time in completing the survey. The survey was administered online and was fielded from March 26, 2015, to May 26, 2015. By opting into to the survey, respondents provided consent to use their anonymous responses to the survey questions. Because this study did not involve patients or patient data, Institutional Review Board approval and patient consent were not required.

### Fertility treatment physician practice survey

A survey was developed to assess current fertility treatment practices, with a focus on ART procedures. The survey was pre-tested through interviews and consultations with 33 fertility specialists throughout 21 countries. The online questionnaire used in the current study included both quantitative and qualitative questions. Quantitative questions covered the following topics: the number of patients having difficulty conceiving; the main causes for fertility issues; and for each age group split, the number of cycles performed, the type of ART procedure used, and the ART treatment outcomes. ART treatment outcomes were assessed using 4 outcome metrics: the proportion of cycles for which an embryo reached the developmental stage sufficient for the embryo to be transferred, the implantation rate, the proportion of cycles that resulted in pregnancy (i.e., PR) and the proportion of cycles that resulted in a live birth (i.e., LBR). Qualitative questions covered trends in diagnosis, unmet needs in the field of fertility treatment, the perceived biggest improvement in fertility treatment in the past 30 years, and expected development(s) that could be the next “game changer” in fertility treatment. All respondents provided qualitative comments because these sections of the survey were mandatory.

### Data analysis

All survey data were analyzed in aggregate and the individual identities of the survey respondents were blinded to the study authors. The planned analyses for quantitative data were descriptive and included means and percentages. Qualitative data were analyzed thematically and coded according to the main themes of the survey questions. Any response that addressed multiple themes was counted as multiple comments.

## Results

A total of 1435 physicians responded to the survey invitation and 363 met eligibility criteria and completed the survey. The number of respondents by country who completed the survey included 157 fertility specialists in the EU5 (29 in France, 33 in Germany, 23 in Italy, 38 in Spain, and 34 in the UK), 91 in the US, 50 in China, and 65 in Japan.

### Physicians’ practice

The mean number of women with difficulty conceiving seen per month ranged from 63 in Italy to 236 in China (Table 1). In terms of age split, patients >42 years old represented the lowest proportion of patients seen in all countries, with the exception of Spain, ranging from 6 % in France to 15 % in Italy. In Spain, the lowest proportion of patients seen was within the youngest category of age <35 years old (10 %) followed by patients >42 years old (11 %), and the highest proportion of patients seen occurred in the age category of 35 to 39 years old (63 %).

**Table 1:** Women seen per month by fertility specialists by country and age group

		Percentage of women by age group				
		Number of women (Mean)	<35 y	35 to 39 y	40 to 42 y	>42 y
France	*n* = 29	82.4	37 %	37 %	20 %	6 %
Germany	*n* = 33	175.9	26 %	50 %	16 %	9 %
Italy	*n* = 23	62.6	18 %	43 %	24 %	15 %
Spain	*n* = 38	118.8	10 %	63 %	17 %	11 %
UK	*n* = 34	75.8	30 %	39 %	19 %	12 %
US	*n* = 91	145.3	37 %	33 %	21 %	9 %
China	*n* = 50	235.8	46 %	32 %	14 %	8 %
Japan	*n* = 65	145.4	29 %	39 %	24 %	9 %

*UK* United Kingdom, *US* United States, *Y* years

The main causes for the difficulty conceiving were very similar across all countries, with male factor, both male and female factor, diminished ovarian reserve, and ovulatory dysfunction being the most frequently reported causes of infertility (Table 2). The only meaningful difference among countries was the report of tubal factor as the infertility cause in 36 % of women in China, whereas tubal factor was reported as ≤16 % in all other countries.

**Table 2:** The percentage of fertility specialists reporting the cause of infertility by country

Causes of infertility	France	Germany	Italy	Spain	UK	US	China	Japan
	*n* = 29	*n* = 33	*n* = 23	*n* = 38	*n* = 34	*n* = 91	*n* = 50	*n* = 65
Male factor	27 %	31 %	22 %	24 %	26 %	24 %	20 %	22 %
Female and male factor	25 %	27 %	21 %	29 %	20 %	21 %	17 %	19 %
Diminished ovarian reserve	17 %	16 %	24 %	28 %	17 %	22 %	11 %	18 %
Ovulatory dysfunction	21 %	17 %	14 %	11 %	17 %	23 %	20 %	25 %
Tubal factor	11 %	13 %	11 %	7 %	13 %	11 %	36 %	16 %
Multiple female factor	15 %	17 %	10 %	13 %	10 %	16 %	14 %	19 %
Unknown	13 %	9 %	13 %	13 %	21 %	12 %	6 %	24 %
Endometriosis	11 %	13 %	10 %	13 %	10 %	11 %	15 %	15 %
Uterine factor	5 %	5 %	4 %	5 %	5 %	5 %	10 %	6 %
Stress/Psychological factor	4 %	3 %	3 %	4 %	4 %	2 %	7 %	7 %
Other	3 %	3 %	1 %	2 %	2 %	1 %	4 %	1 %

*UK* United Kingdom, *US* United States

Among the categories of fertility management, ART use was highest in the EU5, ranging from 47 % of fertility specialists in France to 75 % in Spain, compared with 37 % in the US, 38 % in China, and 27 % in Japan (Table 3). Intrauterine insemination/ovulation induction was reported most frequently by fertility specialists in the US (35 %). Use of the drug clomiphene was highest in China and Japan, reported by 22 and 23 % of fertility specialists, respectively, and ranged from 2 % (Spain) to 14 % (US) of fertility specialists in the other countries.

**Table 3:** Percentage of fertility specialists reporting use of fertility management treatments

Fertility management types	France	Germany	Italy	Spain	UK	US	China	Japan
	*n* = 29	*n* = 33	*n* = 23	*n* = 38	*n* = 34	*n* = 91	*n* = 50	*n* = 65
IUI/OI	27 %	19 %	22 %	16 %	11 %	35 %	14 %	27 %
ART	47 %	49 %	56 %	75 %	59 %	37 %	38 %	27 %
Clomiphene	6 %	13 %	7 %	2 %	10 %	14 %	22 %	23 %
Watch and wait	10 %	7 %	9 %	4 %	12 %	4 %	15 %	14 %
Surgery	9 %	9 %	5 %	3 %	7 %	8 %	9 %	4 %
Other	1 %	3 %	0 %	0 %	1 %	2 %	2 %	4 %

*ART* assisted reproductive technology, *IUI/OI* intrauterine insemination/ovulation induction *UK* United Kingdom, *US* United States

### Patient volume

**Fig. 1:**
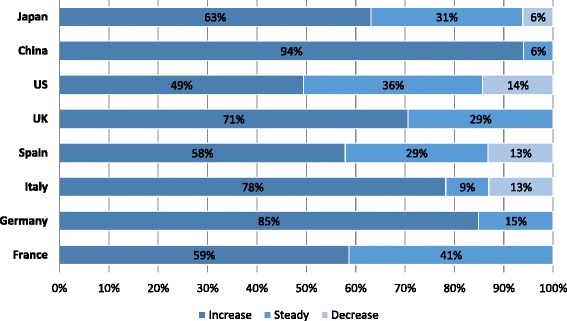
Trends in fertility specialist patient volume. Trends in fertility specialist patient volume are shown by country for women with difficulty conceiving over the prior 2-year period. The percentage of fertility specialist respondents who reported an increased volume, steady volume, or decreased volume is shown within the bars of the figure. UK, United Kingdom; US, United States

The number of women who consulted for issues related to difficulty conceiving increased over the prior 2 years according to 67 % (242/363) of the fertility specialists. The percentage of survey respondents who reported an increase in patient volume ranged from 49 % in the US to 85 % in Germany and 94 % in China (Fig. 1). Fertility specialists in France, Germany, Italy, Spain and Japan most frequently reported the aging population as the reason for the increase in patient volume. In the UK and the US, survey respondents reported the increase in patient volume was driven by increased referrals and improved access to IVF centers. Fertility specialists in China reported an increased number of infertility cases due to pollution, irradiation, and/or lifestyle habits. A decrease in the number of women who consulted for issues related to difficulty conceiving was reported in Italy, Spain, the US, and Japan by 6.9 % (25/363) of the fertility specialists. Respondents from Italy and Spain reported the decrease was due to the economic crisis. In the US and Japan, the decrease was reported as being due to increased availability of clinics providing fertility services and a reduction in financial coverage for patients.

### Current ART practice

**Fig. 2:**
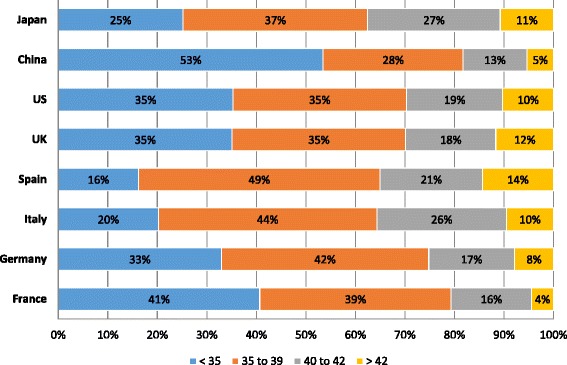
The proportion of patients who received ART procedures by age group. Fertility specialists reported the percentage of their patients who received ART procedures across the patient age groups <35 years old, 35 to 39 years old, 40 to 42 years old, and >42 years old. The percentages are shown by country and by age within the bars of the figure. ART, assisted reproductive technology; UK, United Kingdom; US, United States

The mean number of ART procedures performed per year greatly varied across countries. The fertility specialists based in Japan, Italy, and the US had the lowest mean number of ART procedures at 170, 242, and 253 per year, respectively, followed by fertility specialists based in Spain (341 procedures), France (353), Germany (462) and the UK (492). Fertility specialists based in China had the highest mean number of ART procedures per year at 894. The proportion of ART procedures performed by age group is shown in Fig. 2. A high proportion of ART procedures were done for patients <35 years old in China (53 %), whereas a low proportion of ART procedures were done for patients <35 years old in Spain (16 %). The majority of patients completed 1 to 3 ART cycles with a slight trend toward more ART cycles as patient age increased (Table 4).

**Table 4:** Percentage of patients who received from 1 to >3 ART cycles by patient age group

		<35 years old				35 to 39 years old				40 to 42 years old				>42 years old			
		ART cycles				ART cycles				ART cycles				ART cycles			
		1	2	3	>3	1	2	3	>3	1	2	3	>3	1	2	3	>3
France	*n* = 29	36 %	27 %	20 %	18 %	34 %	28 %	21 %	17 %	36 %	29 %	19 %	16 %	40 %	22 %	16 %	22 %
Germany	*n* = 33	38 %	36 %	18 %	9 %	24 %	37 %	27 %	12 %	18 %	29 %	24 %	28 %	21 %	28 %	37 %	15 %
Italy	*n* = 23	54 %	26 %	15 %	4 %	33 %	44 %	18 %	5 %	28 %	46 %	17 %	8 %	32 %	45 %	11 %	12 %
Spain	*n* = 38	59 %	24 %	11 %	6 %	54 %	25 %	12 %	9 %	46 %	32 %	14 %	8 %	50 %	33 %	11 %	6 %
UK	*n* = 34	53 %	28 %	13 %	6 %	46 %	32 %	14 %	8 %	42 %	32 %	16 %	10 %	49 %	28 %	16 %	7 %
US	*n* = 91	57 %	26 %	13 %	4 %	44 %	32 %	19 %	5 %	35 %	31 %	21 %	13 %	40 %	28 %	21 %	11 %
China	*n* = 50	57 %	26 %	12 %	6 %	39 %	32 %	18 %	11 %	26 %	29 %	23 %	22 %	25 %	26 %	26 %	23 %
Japan	*n* = 65	33 %	28 %	20 %	20 %	27 %	26 %	26 %	22 %	23 %	24 %	23 %	30 %	24 %	21 %	18 %	37 %

*ART* assisted reproductive technology, *UK* United Kingdom, *US* United States

Survey respondents reported the percentage of their patients who received ICSI, fresh embryo, and donor embryo ART cycles (Table 5). ICSI was most frequently performed in Spain, Italy, Germany, France, and the US where it was used in 55 to 89 % of cycles. ICSI was used slightly less frequently by fertility specialists based in the UK, China, and Japan, ranging from 35 to 56 % of ART cycles. With the exception of Italy, all countries showed an increase in the frequency of ICSI cycles with increased patient age. Fresh embryos were largely used by fertility specialists based in France, Germany, and Italy, ranging from 65 to 94 % of ART cycles. In these countries, the use of fresh embryos increased with increased patient age. Among fertility specialists based in Spain, the UK, and the US, there was little variation in the use of fresh embryos by patient age group. Use of fresh embryos fluctuated between 68 to 75 % in Spain, 72 to 83 % in the UK, and 67 to 73 % in the US across patient age groups. In China, the opposite trend was reported with the use of fresh embryos decreasing with increased patient age, ranging from 58 % for patients <35 years old to 38 % for patients >42 years old. Fertility specialists based in Japan reported the lowest use of fresh embryos at 36 to 46 % of patients, with little variation by patient age group. Reported use of donor eggs in ART cycles was very limited in France, Italy, and Japan (≤18 %) with no such reports from survey respondents in Germany because use of donor eggs is illegal in that country. Survey participants based in China used donor eggs in 10 to 20 % of ART cycles. Fertility specialists based in the US and UK reported similar use of donor eggs in ART cycles, ranging from 7 and 9 % for patients <35 years old to 45 % and 46 % for patients >42 years old, respectively. Fertility specialists based in Spain reported the most frequent use of donor eggs, ranging from 11 % for patients <35 years old to 58 % for patients >42 years old.

**Table 5:** Percentage of patients who received type of ART treatment by patient age group

		Proportion of ICSI cycle per age group (y)				Proportion of fresh embryo cycle per age group (y)				Proportion of donor cycle per age group (y)			
		<35	35 to 39	40 to 42	>42	<35	35 to 39	40 to 42	>42	<35	35 to 39	40 to 42	>42
France	*n* = 29	55 %	55 %	58 %	60 %	65 %	72 %	85 %	92 %	3 %	4 %	7 %	10 %
Germany	*n* = 33	62 %	67 %	71 %	75 %	73 %	76 %	83 %	94 %	0 %	0 %	0 %	0 %
Italy	*n* = 23	80 %	74 %	78 %	86 %	76 %	78 %	87 %	89 %	1 %	2 %	5 %	18 %
Spain	*n* = 38	70 %	77 %	89 %	89 %	71 %	75 %	72 %	68 %	11 %	27 %	49 %	58 %
UK	*n* = 34	48 %	51 %	54 %	56 %	72 %	74 %	83 %	81 %	9 %	12 %	23 %	45 %
US	*n* = 91	68 %	71 %	75 %	76 %	69 %	67 %	69 %	73 %	7 %	11 %	24 %	46 %
China	*n* = 50	35 %	41 %	48 %	51 %	58 %	56 %	48 %	38 %	10 %	10 %	19 %	20 %
Japan	*n* = 65	40 %	45 %	49 %	52 %	39 %	36 %	43 %	46 %	11 %	3 %	2 %	2 %

*ART* assisted reproductive technology, *ICSI* intracytoplasmic sperm injection, *UK* United Kingdom, *US* United States, *Y* years

The number of embryos, categorized as single embryo transfer (SET), dual embryo transfer (DET), and >2 embryos transferred (>DET) back to the patient at each ART cycle increased with increased patient age (Table 6). The majority of patients <35 years old received SET or DET across all countries. Among patients >42 years old, 41 % of patients in the US received > DET, 30 % of patients in Italy received > DET, and 17 to 18 % of patients in France, Germany, and the UK received > DET. In China, Spain, and Japan, ≤9 % of patients >42 years old received > DET.

**Table 6:** Percentage of patients who received single, dual, or more than 2 embryo transfers by patient age group

		<35 years old			35 to 39 years old			40 to 42 years old			>42 years old		
		SET	DET	> DET	SET	DET	> DET	SET	DET	> DET	SET	DET	> DET
France	*n* = 29	54 %	45 %	1 %	39 %	58 %	3 %	19 %	71 %	10 %	15 %	68 %	17 %
Germany	*n* = 33	18 %	80 %	2 %	16 %	74 %	11 %	16 %	67 %	17 %	30 %	53 %	17 %
Italy	*n* = 23	35 %	61 %	4 %	24 %	53 %	23 %	24 %	42 %	34 %	34 %	37 %	30 %
Spain	*n* = 38	46 %	52 %	2 %	35 %	58 %	6 %	39 %	56 %	5 %	55 %	42 %	3 %
UK	*n* = 34	77 %	23 %	0 %	62 %	38 %	0 %	39 %	46 %	14 %	41 %	41 %	18 %
US	*n* = 91	42 %	56 %	1 %	30 %	62 %	8 %	14 %	52 %	33 %	16 %	43 %	41 %
China	*n* = 50	28 %	70 %	3 %	26 %	68 %	6 %	28 %	66 %	6 %	24 %	67 %	9 %
Japan	*n* = 65	91 %	7 %	2 %	86 %	13 %	1 %	69 %	29 %	2 %	71 %	26 %	3 %

*DET* dual embryo transfer, >*DET* more than 2 embryos transferred, *SET* single embryo transfer, *UK* United Kingdom, *US* United States

### ART procedure outcomes

**Fig. 3:**
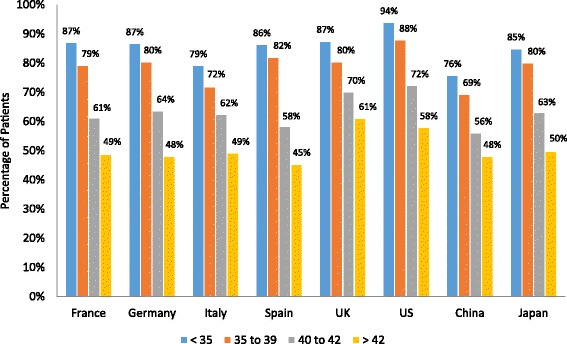
Percentage of patients with implantable embryos. Fertility specialists reported the percentage of their patients who had implantable embryos following an ART procedure. Results are reported by country and by patient age group. ART, assisted reproductive technology; UK, United Kingdom; US, United States

**Fig. 4:**
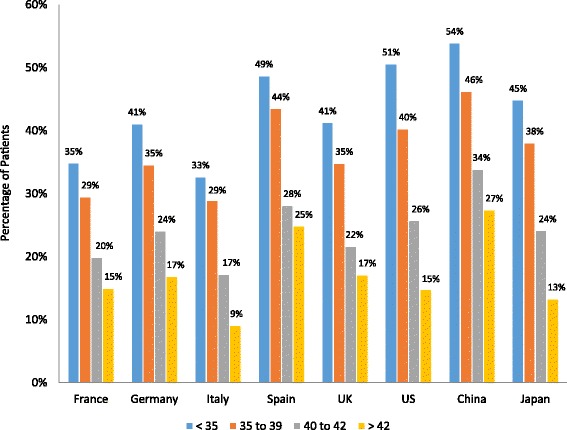
Implantation rate by patient age group. Fertility specialists reported the percentage of their patients who had implantation of an embryo following an ART procedure. Results are reported by country and by patient age group. ART, assisted reproductive technology; UK, United Kingdom; US, United States

**Fig. 5:**
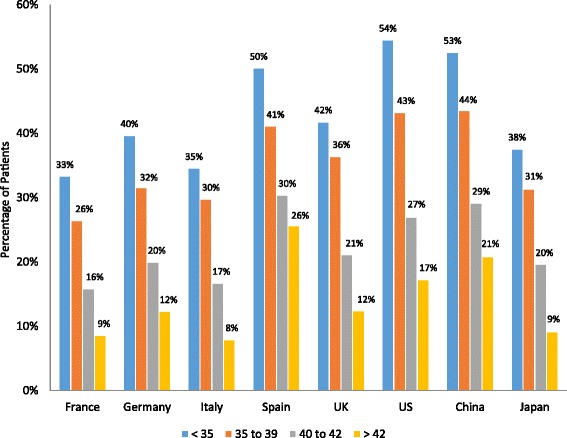
Pregnancy rate by patient age group. Fertility specialists reported the percentage of their patients who had a pregnancy following an ART procedure. Results are reported by country and by patient age group. ART, assisted reproductive technology; UK, United Kingdom; US, United States

**Fig. 6:**
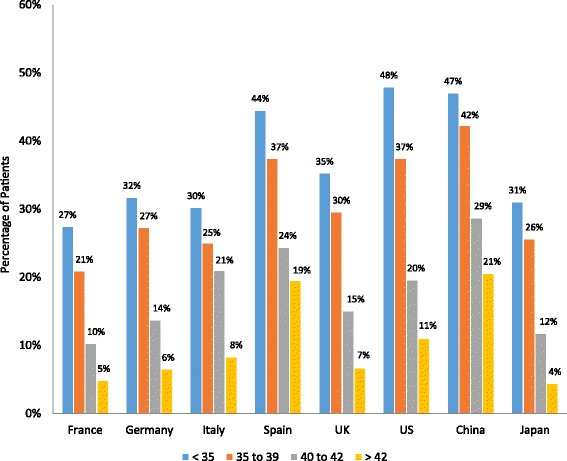
Live birth rate by patient age group. Fertility specialists reported the percentage of their patients who had a live birth outcome following an ART procedure. Results are reported by country and by patient age group. ART, assisted reproductive technology; UK, United Kingdom; US, United States

All 4 ART procedure outcome measures, including percentage of patients with implantable embryos, implantation rate, PR, and LBR, declined with age in all countries (Figs. 3, 4, 5 and 6). The percentage of patients with implantable embryos was similar across all countries and ranged from an average of 85 % for patients <35 years old to 51 % for patients >42 years old (Fig. 3). Among patients <35 years old, the fertility specialists based in China reported the highest implantation rate (54 %), while the lowest implantation rates were reported by specialists based in Italy and France (33 and 35 %, respectively) (Fig. 4). The implantation rate among patients >42 years old ranged from 9 % of patients in Italy to 25 % of patients in Spain and 27 % of patients in China. Among patients <35 years old, the reported PR ranged from 33 % of patients in France to 53 % of patients in China and 54 % in the US (Fig. 5). For patients >42 years old, the PR ranged from 9 % of patients in France and Japan to 26 % of patients in Spain. The lowest LBR among patients <35 years old was reported by fertility specialists in France at 27 % of patients (Fig. 6). Specialists in China and the US reported an LBR of 47 and 48 %, respectively, in this patient age group. For patients >42 years old, the reported LBR ranged from 4 to 6 % of patients in Japan, France, and Germany, up to 19 % of patients in Spain, and 21 % in China.

### Important improvements in fertility treatments

**Fig. 7:**
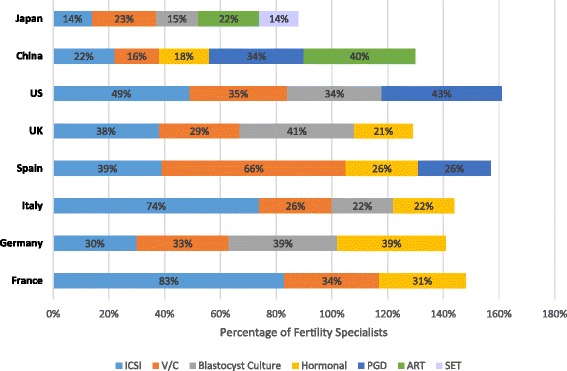
Most important improvements in ART fertility treatment within the past 30 years. Fertility specialists reported their perceived most important improvements in the field of ART fertility treatment within the past 30 years. Results are shown as the percentage of fertility specialists reporting an improvement by country. The percentages of fertility specialists are shown within the bars. The reporting specialists within a country could overlap among the improvement categories and total >100 % as specialists were allowed to report >1 important improvements in fertility treatment. ART, assisted reproductive technology; ICSI, intracytoplasmic sperm injection; PGD, preimplantation genetic diagnosis; SET, single embryo transfer; UK, United Kingdom; US, United States; V/C, vitrification and cryopreservation

Survey participants identified what they perceived to be the greatest improvements in the field of fertility treatment within the past 30 years. The relative rankings of the improvements varied by country. However, improvements that were most frequently cited included ICSI (ranging from 14 % of respondents in Japan to 83 % in France), vitrification and cryopreservation techniques that provide the ability to freeze gametes and embryos (ranging from 16 % of respondents in China to 66 % in Spain), improvement in hormonal drugs and treatment protocols (ranging from 18 % of respondents in China to 39 % in Germany), and the ability to culture embryos up to the blastocyst stage (ranging from 15 % of respondents in Japan to 41 % in the UK) (Fig. 7). PGD was mentioned as a major improvement by fertility specialists in only 3 countries: the US (43 %), Spain (26 %), and China (34 %). Less frequently reported across all countries was the development of ART itself (22 % of respondents in Japan and 40 % of respondents in China) and SET (14 % of respondents in Japan).

### Unmet needs and expected advancements in fertility treatment

**Fig. 8:**
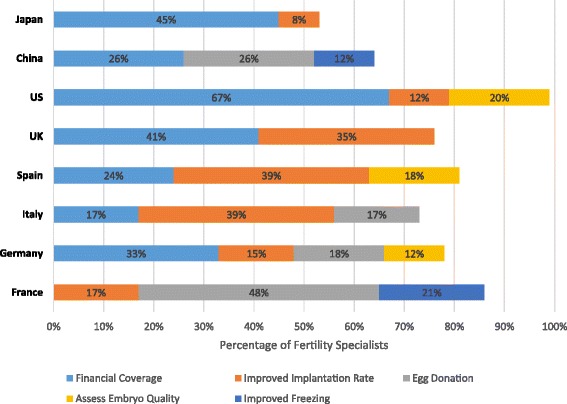
Greatest unmet needs in current ART fertility treatment. Fertility specialists reported their perceived current greatest unmet needs in the field of ART fertility treatment. Results are shown as the percentage of fertility specialists reporting an unmet need by country for the top 5 reported unmet needs. The percentages of fertility specialists are shown within the bars. The reporting specialists within a country could overlap among the unmet needs categories as specialists were allowed to report >1 unmet need in fertility treatment. ART, assisted reproductive technology; UK, United Kingdom; US, United States

Fertility specialists reported current unmet needs for patients who have difficulty conceiving and choose to pursue fertility treatment. Among the most frequently reported unmet needs, better financial coverage was mentioned by survey respondents from all countries but France, ranging from 17 % of survey respondents in Italy to 67 % in the US (Fig. 8). With the exception of China, improvement in implantation rate was reported as an important unmet need by fertility specialists from all countries, ranging from 8 % of respondents from Japan to 39 % of respondents from Italy and Spain. Egg donation was reported as an important unmet need by specialists from 4 countries: China (26 %), France (48 %), Germany (18 %), and Italy (17 %). Improved ability to preserve eggs, embryos, and sperm through freezing was reported as an unmet need by fertility specialists in China (12 %) and France (21 %) and improved ability to assess the quality and viability of an embryo was reported as an unmet need by specialists in Germany (12 %), Spain (18 %), and the US (20 %). Other unmet needs also reported included improved access to fertility clinics (14 %) and surrogate pregnancy (12 %) by specialists in China and improved counseling and awareness of fertility issues (8 %) and genetic testing (6 %) by specialists in Japan.

**Fig. 9:**
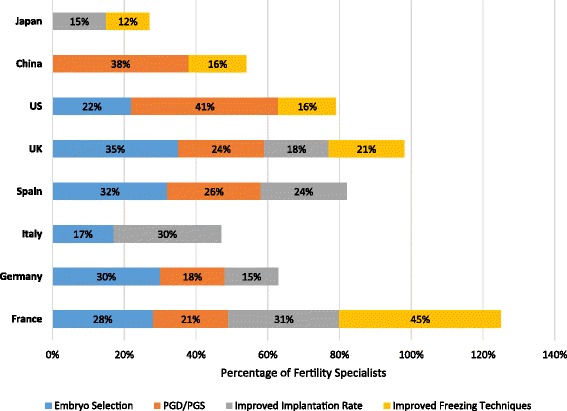
Anticipated “game changing” developments in ART fertility treatment. Fertility specialists reported their anticipated future “game changing” developments in ART fertility treatment. Results are shown as the percentage of fertility specialists reporting an anticipated development by country and the percentages are shown within the bars. The reporting specialists within a country could overlap among the anticipated development categories because specialists were allowed to report >1 anticipated development within fertility treatment. Embryo selection refers to improved selection through imaging and/or metabolomics. ART, assisted reproductive technology; PGD/PGS, preimplantation genetic diagnosis/preimplantation genetic screening; UK, United Kingdom; US, United States

The future advancements that the fertility specialists most frequently reported they expect to occur that could change the field of fertility treatment included improved embryo selection through imaging and/or metabolomics, greater use of PGD and PGS, and development of treatments that improve the implantation rate (Fig. 9). Fertility preservation through further development of freezing techniques for gametes was mentioned by fertility specialists from 5 countries: China (16 %), France (45 %), Japan (12 %), the UK (21 %), and the US (16 %). Other expected improvements that were less frequently reported by the survey respondents were better hormonal treatments (10 % of respondents in China), better understanding of the role of the uterus (24 % of respondents in France and Spain), improvement of fertility in older patients (23 % of respondents in Japan), improved embryo quality through gene modification (10 % of respondents in China), and the use of stem cells or somatic cells to generate gametes (11 % of respondents in Japan).

## Discussion

There are several key findings from the current survey of fertility specialists in 8 countries. There is consensus among the current survey results, the annual CDC ART registry report, and the annual ESHRE ART report that the number of infertility patients seeking fertility treatment is increasing and it is expected that the number of patients will continue to increase due to aging populations [15, 16]. The causes of infertility appear to be universal, with the exception that in China tubal factor was reported as the most important cause of infertility, as compared to tubal factor being in the 5^th^ position in the other countries. As expected, ART outcomes decreased with increased patient age. There is a strong unmet need to develop techniques that will help preserve or restore fertility for older patients. The cost and/or absence of financial reimbursement for IVF fertility treatment appears to be an important barrier to patients’ access to fertility treatment.

ART outcome was reported to be the most successful in Spain, the US, and China. These countries also reported that PGD/PGS was one of the most important fertility treatment improvements in the past year, and this could potentially explain why these countries have better outcomes results. However, many other dimensions that have an effect on the outcomes of fertility treatment should be considered. Thus, the highest success rates in Spain, the US, and China cannot be driven only by the use of PGD/PGS. The number of embryos transferred, the use of fresh versus frozen embryos, as well as other environmental and psychological elements not measured in the current survey can also have an effect on clinical outcomes. The main unmet need reported by fertility specialists is better coverage for the cost of IVF. Fertility treatment is a highly technological field and is very costly, indicating it may be difficult to address this unmet need. Because ART is not fully reimbursed in the studied countries -- with the exception of France -- it is up to the leadership of the clinics to reduce the costs of the procedures.

In terms of future development, the fertility specialists reported that improved embryo selection and implantation rates are expected to change the field of fertility treatment. There appears to be little room for improvement in the hormonal treatment used to produce eggs and several techniques are available to produce embryos, including IVF, ICSI, IMSI, and donor eggs. With the advent of time-lapse monitoring of embryo development and PGD/PGS procedures, embryo selection is one of the newest areas of development in fertility treatment. However, these procedures are approved in only a few countries and have raised some ethical discussions in most. The factors underlying the implantation rate of embryos are not well understood, specifically, there is a need to better understand why good embryos do not successfully implant. Finally, the survey did not attempt to find the root causes of infertility in order to move away from palliating the consequences of infertility and toward the prevention and best treatment of infertility.

The current study survey results compare favorably with and further extend the findings within the most recently reported CDC ART National Summary Report [16]. The CDC report is based on 456 fertility clinics within the US, whereas the current study survey includes only 91 US clinics [16]. The CDC reports ART efficacy for fresh, non-donor eggs, or embryos compared with the current study report of all procedures combined. However, fresh, non-donor embryos represent the majority of procedures in the current study, supporting the comparability between the reports. The fertility treatment outcomes data as measured by embryo transfers, PR, and LBR reported in the current survey of fertility specialists in the US are similar to the CDC registry report data. The comparison suggests that ART efficacy is stable, with no shift in fertility treatment having had an impact on efficacy of treatment, which is consistent with the lack of any major improvements in ART procedures since 2012. The comparison of the current study survey data with the CDC registry report shows that the number of ICSI procedures has increased from 68 % reported by the most recent CDC registry report in 2012 to 73 % reported in 2015 in the current survey (ranging from 68 % in patients <35 years old to 76 % in patients >42 years old). The reported main causes of infertility remained the same, with male factor, ovulatory dysfunction, diminished ovarian reserve, and both female and male factor being the most common causes of infertility, as reported by both the CDC registry report examining 2012 data and the current survey. When comparing the number of embryo transfers with the CDC 2012 registry data report, there was a trend toward more frequent use of single embryo transfer in the current survey.

The comparison of the current study survey results among European countries, China, and Japan with existing registry data is more challenging. The ESHRE registry report (2010) is based on 104 fertility clinics in France, 114 clinics in Germany, 202 clinics in Italy, 103 clinics in Spain, and 72 clinics in the UK [15]. The current survey report includes 29 fertility clinics in France, 33 in Germany, 23 in Italy, 38 in Spain and 34 in the UK. The comparison of outcomes data is complicated because the ESHRE registry reports outcomes data using a different breakdown in the number of cycles, presenting ART outcomes results per type of procedure and per age group; whereas the current study survey split the data only per age group [15, 17]. The number of embryos transferred is more easily compared between the studies, and as was found in comparison with the CDC 2012 report, a trend was observed toward more frequent single embryo transfer in the current survey than in the ESHRE 2010 report. To our knowledge, this is the first report on fertility treatment in China and Japan, therefore no comparison of the current survey results with registry or other data from these countries is possible.

It should be noted that this research has a number of limitations. As with any survey, our findings may be influenced by the recall and response bias of the surveyed individuals. Additionally, only a subset of fertility specialists participated in our survey, and as with all analyses, caution should be used when generalizing results to an entire population. However, the response rate obtained in our survey is in line with what would be expected for this type of survey [18]. Regarding the recruitment of participants, two approaches were used: in the US, France, Germany, Italy, Spain, and the UK, fertility specialists were contacted directly by the Deerfield Institute, and were recruited by e-mail or postal mail. In China and Japan, survey participants were recruited using a panel provider (i.e., the M3 panel) and were invited by e-mail. This might include a bias as the M3 panel includes only physicians who accepted to be part of the panel, while the Deerfield Institute had access to the full population of fertility specialists. It is important to note that all respondents answered the same questions and there was no difference in the questionnaire outside of translation whether respondents were recruited by the Deerfield Institute or the M3 panel. Although patient demographic (e.g., age, smoking, obesity) and biochemical (e.g., hormonal status) characteristics may play a role in fertility treatment outcomes, such patient-level data was outside the scope of the current survey. Future research is needed to examine the influence of patient-level characteristics as well as the patient assessment and preparation protocols used by fertility specialists prior to treatment to ascertain the similarities and differences in global outcomes.

## Conclusions

The survey results provide a unique and comprehensive interim look at the fertility treatment field ahead of the next annual release of the ESHRE and CDC ART publications. The survey results identified a trend toward more frequent single embryo transfer, most likely due to improved identification of viable embryos resulting in an increased chance of pregnancy per embryo transfer. The survey also highlights differences and similarities among countries practicing fertility treatment. As reported by the fertility specialists who participated in the survey, the key important avenues that are expected to change in fertility treatment in the future are the improvement of embryo selection and the improvement of implantation rate.

## Abbreviations

ART: assisted reproductive technology
CDC: Centers for Disease Control and Prevention
DET: dual embryo transfer
>DET: more than 2 embryos transferred
ESHRE: European Society of Human Reproduction and Embryology
EU5: European Union Five
ICSI: intracytoplasmic sperm injection
IMSI: intracytoplasmic morphologically selected sperm injection
IUI: intrauterine insemination
IVF: in vitro fertilization
LBR: live birth rate
OI: ovulation induction
PGD: preimplantation genetic diagnosis
PGS: preimplantation genetic screening
PR: pregnancy rate
SET: single embryo transfer
V/C: vitrification and cryopreservation
UK: United Kingdom
US: United States
